# Changes in the nitrate assimilation pathway serve as a component of maize stress response to competition cues

**DOI:** 10.3389/fpls.2025.1617659

**Published:** 2025-09-09

**Authors:** William Kramer, Sasan Amirsadeghi, Andrew McKenzie-Gopsill, Clarence J. Swanton

**Affiliations:** ^1^ College of Agricultural Sciences, Colorado State University, Fort Collins, CO, United States; ^2^ Department of Plant Agriculture, University of Guelph, Guelph, ON, Canada; ^3^ Charlottetown Research and Development Centre, Agriculture and Agri-Food Canada, Charlottetown, PE, Canada

**Keywords:** kin plants, low red to far-red signal, nitrogen assimilation enzymes, RNA-sequencing, shade avoidance syndrome, transporter genes, weed-crop interaction

## Abstract

The impact of plant competition cues on nitrate levels and their assimilation in major crop plants remains largely unknown. This study investigated how low red to far-red (R:FR) light, a signal of plant competition, affects nitrate levels and assimilation in maize and soybean seedlings. Maize and soybean seedlings were exposed to low R:FR light conditions (R:FR ≈ 0.5) that mimicked nearby weeds or artificial sources. Additional treatments included individual soybean seedlings subjected to a soybean canopy. Biochemical assays and RNA-sequencing were used to assess nitrate levels, assimilation-related enzymes, and gene expression. Low R:FR light led to increased leaf nitrate levels in maize by 95% and 52% compared to the weed-free control (R:FR ≈ 2.8), and in soybean by 50% and 63%, while ammonium levels remained unchanged. A 37% increase in leaf nitrate level was also observed in soybean exposed to canopy conditions. In maize, nitrate accumulation was linked to reduced activity of ferredoxin-dependent glutamine:2-oxoglutarate aminotransferase (fd-GOGAT), while activities of nitrate reductase, nitrite reductase, glutamine synthetase, and NADH-GOGAT were unaffected. RNA-sequencing of maize leaves did not show altered expression of tonoplast localized nitrate transporter genes. These findings suggest that low R:FR light, as a plant competition cue, promotes nitrate accumulation in maize and soybean, potentially by altering nitrate assimilation pathways rather than transport or storage. This response may influence crop growth and metabolism under competition stress.

## Introduction

Nitrate, as a major source of mineral nitrogen (N), regulates a wide array of plant processes including seed dormancy and germination, seedling establishment, root system architecture, shoot growth, and flowering time (Vidal et al., 2020). Plants utilize nitrate through the processes of uptake, assimilation, translocation, recycling, and remobilization to support yield (Masclaux-Daubresse et al., 2010). Nitrate uptake occurs via four elaborate processes known as constitutive high- and low-affinity, and inducible high- and low-affinity transport systems (Tsay et al., 2007; Léran et al., 2014). Nitrate assimilation to ammonium then occurs via two enzymatic reactions. Once nitrate is transported inside the cell, the assimilatory nitrate reductase (NR) catalyses the conversion of nitrate to nitrite, which is transported to the plastids either through diffusion of the protonated form (HNO_2_) or active transport of ionic form via the action of various nitrite transporters (Brunswick and Cresswell, 1988; Shingles et al., 1996; Sugiura et al., 2007). Then, nitrite reductase (NiR) catalyses the conversion of nitrite to ammonium. The assimilation of ammonium into amino acids occurs through sequential reactions of glutamine synthetase (GS) and glutamine:2-oxoglutarate aminotransferase (GOGAT; also known as glutamate synthase). The former catalyses the ATP-dependent condensation of glutamate and ammonium to form glutamine and the latter catalyses the transfer of the amide group from glutamine to 2-oxoglutarate to form glutamate using either pyridine nucleotides (NADH/NADPH) or ferredoxin (fd) as reductants (Zayed et al., 2023; Yoneyama and Suzuki, 2020; Krapp, 2015; Sherameti et al., 2002).

In plants, nitrate levels and activities of nitrate assimilation enzymes are regulated by a multitude of both external and internal factors, and in particular light (Bian et al., 2020; Umar and Iqbal, 2007; Krapp, 2015; Lillo, 2008). There is, however, a dearth of knowledge on nitrate assimilation under conditions such as weed-crop competition, where reflected far-red light from neighbouring weeds triggers the undesirable shade avoidance syndrome (SAS) that negatively affects crop yield (Huber et al., 2021). In natural ecosystems, SAS is often viewed as an adaptive response to increase the fitness of individuals through shoot and internode elongation and maximize light capture (Ruberti et al., 2012). Conversely in agricultural systems, crop plants’ expression of the SAS can reduce biomass accumulation and create a maladapted phenotype especially when weeds are removed through active management practices (Schambow et al., 2019; Page et al., 2009; Weinig and Delph, 2001). In this regard, spinach seedlings exposed to a five-day pre-harvest treatment of supplemental FR light (735 nm LED) displayed a 10-fold increase in leaf nitrate concentration compared with the control (Johnson et al., 1999). Earlier studies showed light-dependent induction of NR activity in radish cotyledons and maize seedlings (Beevers et al., 1965). Further, reversible control of NR activity by red (R) and far-red (FR) light was demonstrated in etiolated terminal buds of field peas (Jones and Sheard, 1972), rice seedlings (Sasakawa and Yamamoto, 1979), and squash cotyledons (Rajasekhar et al., 1988). In *Arabidopsis* seedlings, accumulation of NR transcripts in response to FR light corresponded to the phytochrome-mediated very low fluence response, and NR activity was strongly induced by white light (Pilgrim et al., 1993). Phytochrome-mediated NR responses have been reported in several etiolated plant species (Lillo and Appenroth, 2001) including the low fluence response of NR in maize (Sharma et al., 1994; Raghuram and Sopory, 1999). Subsequent studies showed that two downstream components of phytochrome signaling, ELONGATED HYPOCOTYL 5 (HY5) and HY5 homolog (HYH), were indispensable for phytochrome-mediated light induction of NR activity (Jonassen et al., 2008). In tobacco, coaction of nitrate and light was required for higher induction of NiR transcripts and high irradiance response of phytochrome was involved in higher NiR activity (Neininger et al., 1992). In addition, *Arabidopsis* HY5 was essential for high level expression of *NiR1* gene as well as NiR1 activity under nitrogen limited conditions (Huang et al., 2015).

Following nitrate assimilation, light also regulates the activities of enzymes involved in the conversion of nitrate to ammonia. For example, illumination of etiolated maize leaves increased the plastidic glutamine synthetase (GS) and ferredoxin-dependent glutamine:2-oxoglutarate aminotransferase (fd-GOGAT) levels (Sakakibara et al., 1992). Light is required for nitrate induction of fd-GOGAT activity in barley seedlings (Pajuelo et al., 1997), and continuous light increases the transcription and activity of fd-GOGAT in maize, when exposed to nitrate or ammonium in the environment (Suzuki et al., 1996). In addition to fluence rate, shifts in light quality are also known to induce changes in activities of GOGAT enzymes such that red light pulses induce NADH-GOGAT, whereas FR light pulses can induce fd-GOGAT (Hecht et al., 1988).

In addition to external factors, a wide array of internal cellular factors can affect shoot nitrate content. For example, nitrate transporters (NRTs) play pivotal roles in the movement and accumulation of nitrate within plant leaves (Hu et al., 2014). Notably, low-affinity NRT1, high-affinity NRT2, and nitrate and peptide transporter family member NPF2.13 (NRT1.7) are involved in the uptake, transport, and storage of nitrate, which can lead to its accumulation in leaves (Krapp et al., 2014; Dechorgnat et al., 2011; Fan et al., 2009). Chloride channel (CLC) transporters, specifically CLCa (a vacuolar nitrate transporter) plays a crucial role in nitrate accumulation in plant leaves (Hodin et al., 2023; von der Fecht-Bartenbach et al., 2010). Changes in the allocation and distribution of amino acids within the plant by amino acid transporters such as amino acid permeases (AAPs) can also affect overall nitrogen metabolism and nitrate levels in the leaves (Perchlik and Tegeder, 2018). Further, peptide transporters, which are involved in the transport of peptides and other molecules across cell membranes can affect the distribution and allocation of nitrogen within the plants and thereby impact nitrate levels in the leaves (Komarova et al., 2008).

While many environmental factors that influence nitrate levels and its assimilation have been identified, the effects of low R:FR signals emanating from neighbouring weeds or kin plants on the nitrate assimilation pathway in major crop plants remain largely unexplored. In this study, maize and soybean were exposed to biological (neighbouring weeds) and artificial (FR LEDs) sources of low R:FR light to test the hypothesis that, under resource-independent competition, the low R:FR light emitted by neighbouring weeds could affect the nitrate assimilation pathway similar to that triggered by an artificial low R:FR light source. In both plant systems, the results were consistent with the notion that nitrate accumulation is a component of SAS under resource-independent competition. The occurrence of FR light-mediated SAS under weed competition has negative outcomes particularly for crop yield (Huber et al., 2021) and the identification of critical period for weed control (CPWC) is indispensable for the conservation of crop yield potential (Knezevic and Datta, 2015). Our results raise the possibility that in environments with adequate nitrate for growth, the accumulation of nitrate in response to low R:FR signals from neighboring weeds may lead to nitrate limitation, impacting the metabolism, growth, and development of crop plants. This limitation may offer a new perspective on the empirical concept of CPWC.

## Materials and methods

### Plant material and growth conditions

Experimental plants consisted of a hybrid maize (*Zea mays* (L.) CG108 x CG102; University of Guelph) and soybean (*Glycine max* (L.) Merr. *cv* OAC Wallace; University of Guelph). Plants were raised in controlled environment growth chambers (Model CMP 6050 Conviron, Winnipeg, Canada). Growth conditions for maize and soybean were 16 h light/8 h dark, a temperature of 23°C/18°C (light/dark), a growth irradiance of 500-600 µmol photons m^-2^ s^-1^, and a relative humidity of 60%. Overhead radiation was supplied using a combination of white fluorescent tubes and 100 W incandescent bulbs (Sylvania, Washington, USA). Experimental plants were raised under three light treatments consisting of a weed-free control (R:FR ≈ 2.8), a biological low R:FR light (R:FR ≈ 0.5), and an artificial low R:FR light (R:FR ≈ 0.5). The weed-free control, biological low R:FR light, and artificial low R:FR light treatments were set up by inserting plastic tubes (8 × 18 cm, 1 L) in the centre of plastic pots (16 × 15 cm, 3.36 L; Airlite Plastics Company, Omaha, USA). Two and four drainage holes were drilled into the plastic tubes and plastic pots, respectively. For the weed-free control and artificial low R:FR light treatments, the surrounding areas were filled with baked clay granules (Turface Athletics MVP, Profile Products LLC, Buffalo Grove, USA). For the biological low R:FR light treatment, the surrounding areas were filled with a potting mix consisting of peat moss and perlite (Sunshine #4 Aggergate Plus, Sungro Horticulture, Agawam MA) and seeded (≈ 200 g m^-2^) with a commercial grass mixture of 42% red fescue (*Festuca rubra* L.), 34% Kentucky bluegrass (*Poa pratensis* L.), and 24% perennial ryegrass (*Lolium perenne* L.) (The Scotts Company LLC, Marysville, USA). The pots were well watered and fertilized every two weeks as described previously (Tollenaar, 1989). The surrogate grass was allowed to grow for two months to generate a biological source of reflected FR light prior to the start of experiments. Artificial far-red light was supplied using a combination of 13W FR bulbs (Philips, Koninklijke, N.V.) and Ray44 Fluence Bars (Fluence, Texas, USA). Maize and soybean seeds were planted in a potting mix (Sungro, Massachusetts, USA). All seeds were planted in plastic cups (8 × 10 cm W × H; 355 mL; Dart Container Corp, Mason, USA) to a depth of two cm. The plastic cups were inserted in the plastic tubes in each pot. This prevented direct resource competition between the experimental plants and the surrounding grass. Light interference was prevented by placing a corrugated plastic sheet slightly above the plant level between the pots in the control and biological low R:FR light treatments. This did not affect the free upward airflow (1.5 m^3^ min^-1^) across the growth chamber. Treatments were randomized across the growth chamber between each replication. Maize plants were grown for nine days (4-leaf-tip; i.e., Zadoks stage 4) and fertilized five times with 50 mL of full-strength Hoagland solution (Hoagland, 1933) every other day. Soybean plants were grown for 14 days and fertilized once with 50 mL full-strength Hoagland solution. For the intra-specific competition treatment, the experimental set up involved placing a cup filled with the above-mentioned potting mix at the center of a pot. The area surrounding the cup was then filled with the same potting mix to ensure that the soil level matched that inside the cup. Experiments consisted of a no kin treatment and a six kin treatment. In the no kin treatment, one soybean seed was planted in the cup and the area surrounding the cup remained intact. In the six kin treatment, a single soybean seed was planted inside the cup, while six evenly spaced soybean seeds were planted in the surrounding potting mix. The plastic cup served as a barrier to prevent direct root contact between the central and surrounding plants. Soybean plants were grown and fertilized as described earlier. Leaf tissue was consistently harvested for all assays and consisted of maize second leaves (9-day-old), soybean unifoliate leaves (11-day-old), and soybean first trifoliate leaves (14-day-old). Leaf samples were immediately frozen in liquid nitrogen and ground to a fine powder for assays or stored at -80°C for later use. The light spectral composition for all treatments was assessed at plant height. Light quantity and quality, both incoming and reflected, were measured at 10 different points within each treatment using a LI-COR-180 spectrometer (Li-COR Biosciences, Lincoln, NE, USA). For incoming light measurements, the spectrometer was positioned horizontally at plant height, facing the light sources. For reflected light measurements, it was held at the same height but facing the plants. A summary and detailed data on the light spectral composition for these treatments are presented in **Supplementary Table 1**.

### Metabolite assays

All chemicals were purchased from Sigma Aldrich Canada (Oakville, Ontario, Canada) unless otherwise stated. Nitrate was analyzed by an assay based on nitration of salicylic acid (Cataldo et al., 1975) using approximately 0.2 g of frozen ground plant tissue. The spectral absorbance of the complex formed by nitration of salicylic was measured at *A*_410_ against a blank. Nitrate concentration was determined by linear regression using a standard curve of known concentrations of sodium nitrate (0-800 µmol) and expressed as µmol nitrate per g fresh weight. Ammonium was analyzed after the charcoal treatment of acidic leaf extracts (Bräutigam et al., 2007) from approximately 0.2 g of frozen ground plant tissue. The spectral absorbance of the complex formed by the addition of phenol-sodium nitroprusside and sodium hypochlorite-sodium hydroxide solutions were measured at *A*_620_ against a blank. Ammonium concentration was determined by linear regression using a standard curve of known concentrations of ammonium chloride (0-1 mmol) and expressed as µmol ammonium per g fresh weight.

### Enzyme assays

For all enzyme assays, protein was extracted from approximately 0.1 g of frozen ground second leaf using appropriate buffer solutions, and desalted using a Sephadex G25 column (Amersham Biosciences PD-10). Protein was quantified by a modified Lowry assay (Larson et al., 1986) using bovine serum albumin as a standard (0-20 µg/µL). Previously established methods were used for quantification of activities of nitrate reductase, NR, and nitrite reductase, NiR (Wray and Fido, 1990; Ali et al., 2007), glutamine synthetase, GS (Lea et al., 1990), NADH-dependent glutamine:2-oxoglutarate aminotransferase, NADH-GOGAT (Esposito et al., 2005), and ferredoxin-dependent glutamine:2-oxoglutarate aminotransferase, fd-GOGAT (Yang et al., 2016). The activity of NR was determined using a 100 µL aliquot of the protein extract for 20 minutes at 25°C, and pH 7.5. Absorbance was measured at *A*_540_ following a 15-minute incubation of the reaction at 25°C for color development and corrected using a test blank. The amount of nitrite generated during the reaction was calculated by linear regression using a standard curve of sodium nitrite (0-10 nmol). The NR activity was expressed as µM nitrite generated per minute per mg protein. The activity of NiR was determined using a 5 µL aliquot of the protein extract for 10 minutes at 25°C, and pH 7.5. Absorbance was measured at *A*_540_ following a 15-minute incubation of the reaction at 25°C for color development and corrected against a test blank. The amount of nitrite was calculated by linear regression using a standard curve of sodium nitrite (0-10 nmol), and the NiR activity was expressed as µM nitrite consumed per minute per mg protein. The activity of GS was determined using a 200 µL aliquot of the protein extract for 15 minutes at 25°C, and pH 7.8. Absorbance was measured at *A*_540_, corrected against a test blank, and the amount of γ-glutamyl hydroxamate (GGH) was calculated using a standard curve of γ-glutamyl hydroxamate (0-300 µmol). The GS activity was expressed as µmol γ-glutamyl hydroxamate per minute per mg protein. The activity of NADH-GOGAT was determined using a 150 µL aliquot of the protein extract for six minutes at 25°C, and pH 7.5. The rate of NADH oxidation was monitored at *A*_340_ every minute against a control to correct for endogenous NADH oxidation. The NADH-GOGAT activity was expressed as µmol NADH oxidized per minute per mg protein. The fd-GOGAT activity was determined using a 100 µL aliquot of the protein extract for six minutes at 30°C, and pH 8.5. The rate of NADH oxidation was monitored at *A*_340_ every minute, and the ferredoxin-dependent GOGAT activity was verified using a control that contained all the assay components except for ferredoxin. The fd-GOGAT activity was expressed as µmol NADH oxidized per minute per mg protein.

### RNA-sequencing

For each treatment (weed-free, biological low R:FR, and artificial low R:FR light), a pool of the second leaves of maize (9-day-old) from three individual plants were harvested and replicated three times. In total, RNA was extracted from nine pooled samples using TRIzol reagent. The DNase treatment of RNA samples was performed using an RNase-Free DNase Set (Qiagen). The RNA quality and quantity were determined using an Agilent TapeStation 4150 (Agilent Technologies). The RNA libraries were constructed in three replicates with the Illumina TrueSeq RNA kit (Illumina, Inc.) as described in manufacturer’s protocol. Sequencing of the RNA libraries were performed on an Illumina sequencer (NovaSeq 6000) at the Genome Quebec Innovation Center (McGill University, Canada) to obtain ≈25 million reads per replicate. RNA sequencing data were processed using a standardized pipeline. The maize genome assembly and corresponding GFF annotation file were downloaded from Ensembl Plants (2019, Zm-B73-REFERENCE-NAM-5.0). The genome was indexed using the STAR aligner (Dobin et al., 2013, version 2.5.2b) to prepare for read alignment. Raw FASTQ files were preprocessed using Fastp (Chen et al., 2018, version 0.23.2) for quality trimming and adapter removal, ensuring high-quality input reads for downstream analysis. Trimmed reads were aligned to the indexed maize genome using STAR, allowing for multimapped reads to be appropriately handled. The aligner generated BAM files as output, which were subsequently used for quantification. Gene counts were extracted from the BAM files using the featureCounts tool (Liao et al., 2014, 2.0.1), employing the GFF annotation file for feature definitions. The resulting gene count matrix was imported into R (R version 4.2.3) for differential gene expression analysis using the DESeq2 package (Love et al., 2014, version 1.38.3). This analysis pipeline included normalization and statistical testing to identify differentially expressed genes. All software was run using default parameters unless otherwise specified, and custom scripts for data visualization and additional analyses were executed in R. Adjusted *p*-values (*p*adj) were attained within DESeq2 using the Benjamini-Hochberg false discovery rate (FDR) correction procedure and log_2_ fold change (lfc) tables were generated as described previously (Zhu et al., 2019). The RNA-seq raw reads and expression analysis were deposited in the gene expression omnibus (GEO) data repository under accession GSE213949 (https://www.ncbi.nlm.nih.gov/gds/?term=GSE213949).

### Prediction of intracellular localization of proteins

The protein sequences of DEGs encoding thioredoxins (TRXs) were obtained from maize genetics database (MaizeGDB; http://www.maizegdb.org) and the presence of N-terminal pre-sequences directing proteins to different subcellular compartments was predicted using the protein subcellular localization prediction tool WolF PSORT (https://wolfpsort.hgc.jp).

### Statistical analysis

All experiments were arranged as a randomized complete block design with six maize or soybean plants per treatment and three replications per experiment. The main treatments consisted of varying light quality (weed-free control, biological, and artificial low R:FR light). The weed-free control and low R:FR light treatments were considered the fixed effect. The block and interaction between treatment and block were considered random effects. All data were statistically analyzed with generalized linear mixed effects models in SAS v9.4 (SAS Institute, Cary, NC, USA) using the PROC GLIMMIX procedure. Data normality was confirmed using the Shapiro-Wilk test. All data were tested for ANOVA assumptions and least square means. Least squares means were calculated and the means were separated using Tukey’s Honest Significant Difference (HSD) test. The Standard errors were generated with a type 1 error of α = 0.05 to test for significance among treatments.

## Results

### Low R:FR light environments increase nitrate levels but does not affect ammonium levels in maize leaves

**Figure 1:**
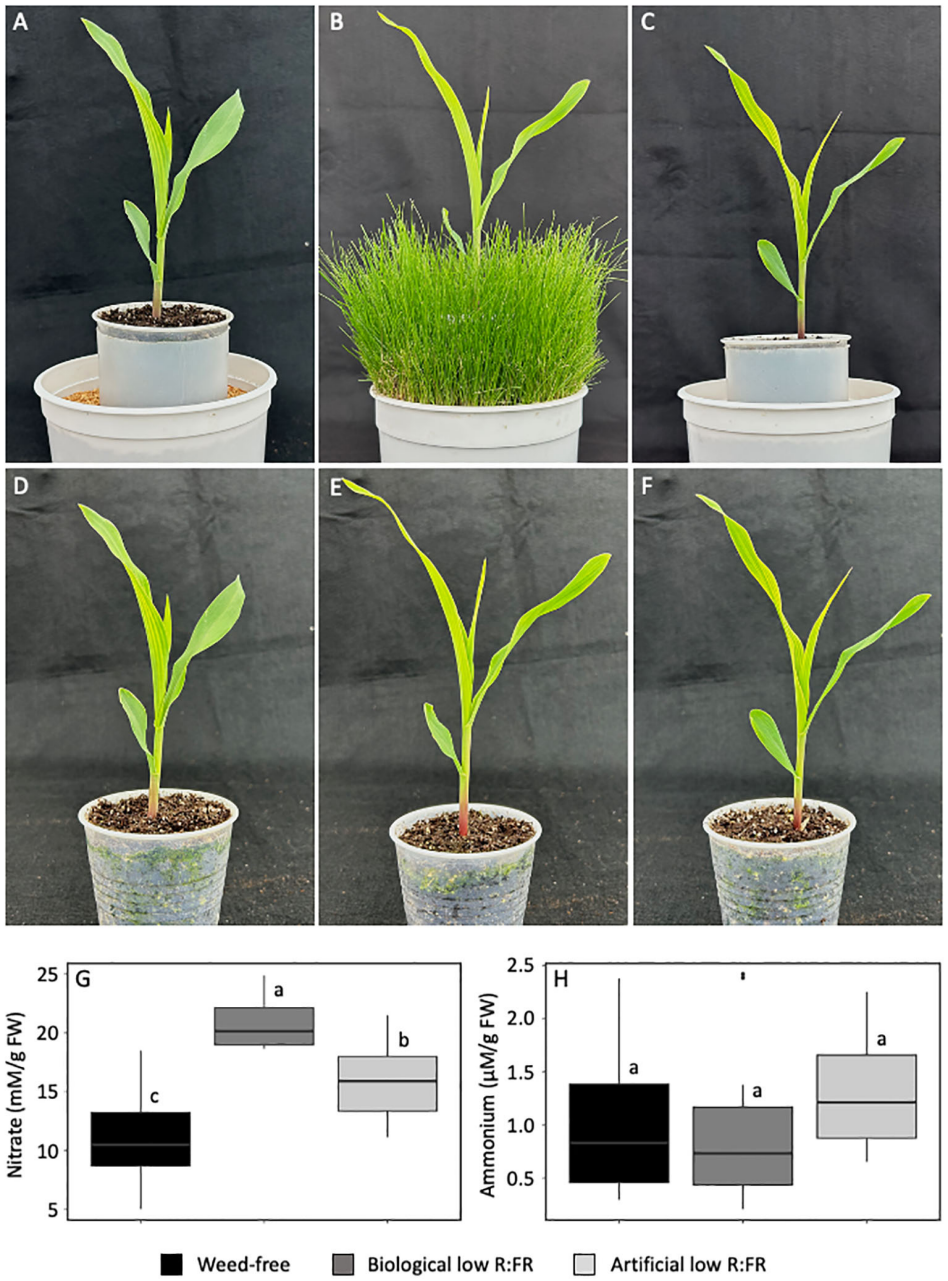
Maize seedlings (9-day-old) in the weed-free control **(A)** biological low R:FR light **(B)** and artificial low R:FR light **(C)** treatments with R:FR ≈ 2.8, ≈ 0.5, and ≈ 0.5, respectively. Plastic cups containing maize seedlings in the weed-free control **(D)** biological low R:FR light **(E)** and artificial low R:FR light **(F)** treatments were uniformly placed in the plastic tubes in the center of the pots, which prevented resource competition between maize and neighbouring weeds in the biological low R:FR light treatment. Increases in leaf nitrate levels **(G)** and no change in leaf ammonium levels **(H)** in the biological and artificial low R:FR light treatments. The data are presented as least square means ± SE from three independent experiments, each with six plants per treatment. Means were compared using Tukey’s HSD test (*p*<0.05). Letters denote statistically significant differences among treatments.

We investigated whether nitrate accumulation can occur in maize leaves exposed to low R:FR light from neighbouring weeds similar to that reported previously in spinach leaves (Johnson et al., 1999). We included in our experiments an artificial source of FR light and performed our experiments under resource-independent competition, where competition for incoming light, water and nutrients were excluded (**Figures 1A-F**). The biological and artificial low R:FR light treatments increased nitrate levels in maize leaves by 95% and 52% (*p*<0.05), respectively, compared with the control treatment (**Figure 1G**). In contrast, ammonium levels in the biological and artificial low R:FR treatments did not differ from that in the control treatment (**Figure 1H**). These results demonstrate that the reflected FR light from neighbouring weeds can indeed increase nitrate levels in maize leaves. Further, the increase in nitrate levels and no change in ammonium levels under the low R:FR light treatments indicate the specificity of this low R:FR light response.

### Increase in leaf nitrate concentration occurs in soybean in response to low R:FR light

**Figure 2:**
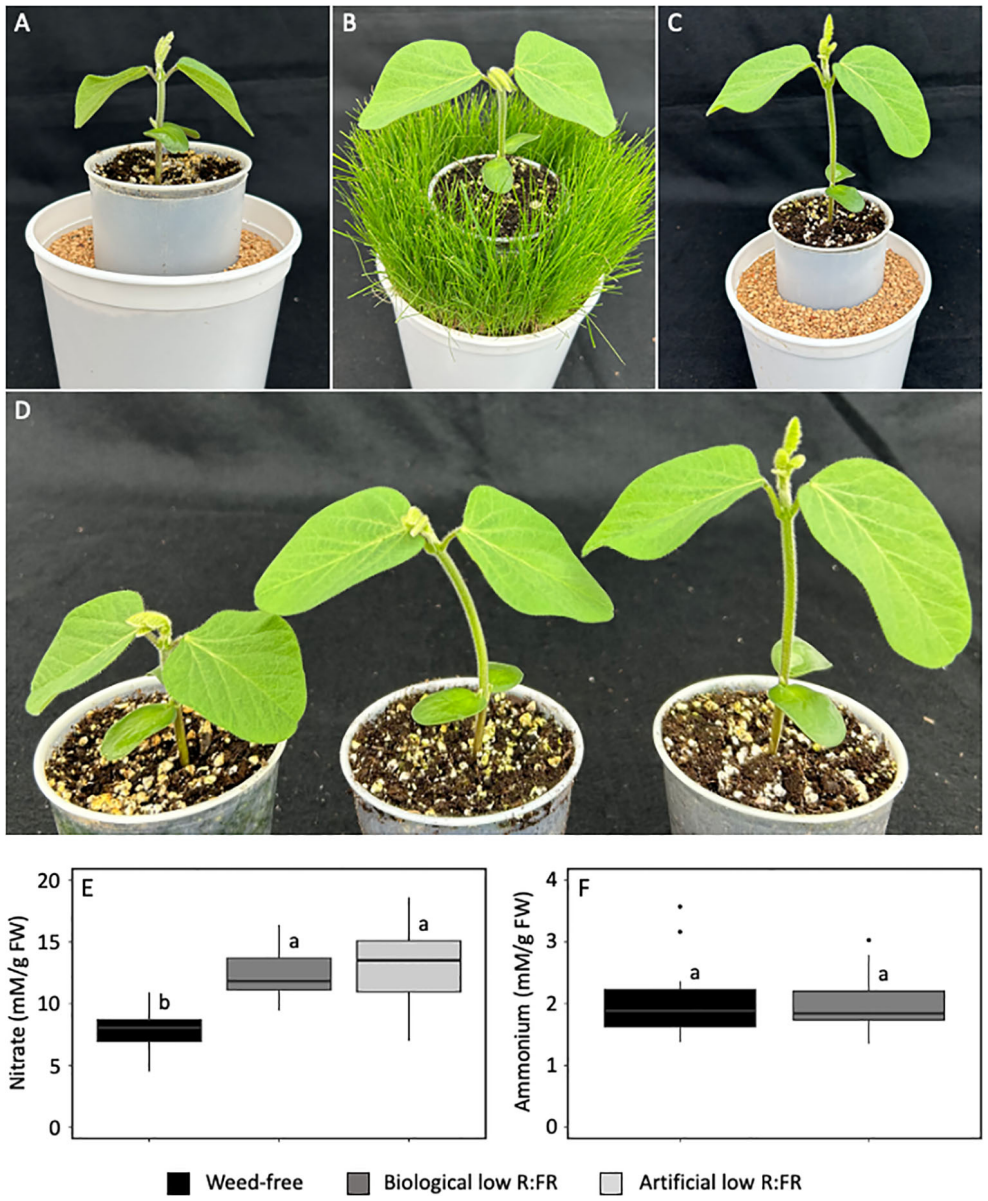
Soybean seedlings (11-day-old) in the weed-free control **(A)** biological low R:FR light **(B)** and artificial low R:FR light **(C)** treatments with R:FR ≈ 2.8, ≈ 0.5, and ≈ 0.5, respectively. Plastic cups containing soybean seedlings **(D)** show normal growth in the weed free treatment (left) and elongation growth in response to low R:FR in the biological low R:FR light (middle), and artificial low R:FR light (right) treatments. Increases in leaf nitrate levels in the biological and artificial low R:FR light treatments **(E)** and no change in leaf ammonium level **(F)** in the biological low R:FR treatment. The data are presented as least square means ± SE from three independent experiments, each with six plants per treatment. Means were compared using Tukey’s HSD test (*p*<0.05). Letters denote statistically significant differences among treatments.

Soybean seedlings were grown in the weed-free control, biological low R:FR, and artificial low R:FR light treatments (**Figures 2A-D**), and the levels of nitrate and ammonium were measured in unifoliate leaves to investigate the differences between the responses of maize and soybean in the low R:FR light treatments. In a similar manner to maize, nitrate levels in soybean unifoliate leaves were increased by 50% in the biological low R:FR treatment and 63% in the artificial low R:FR treatment relative to the control (*p*<0.05) (**Figure 2E**). In addition, the biological low R:FR treatment did not affect ammonium levels in soybean unifoliate leaves (**Figure 2F**), which was consistent with our findings in maize leaves. Overall, these findings along with the earlier report of nitrate accumulation in spinach leaves under supplemental FR light (Johnson et al., 1999) suggest that nitrate accumulation may occur in a wider range of plant species under low R:FR light environments.

### Increase in leaf nitrate concentration occurs under intra-specific competition

**Figure 3:**
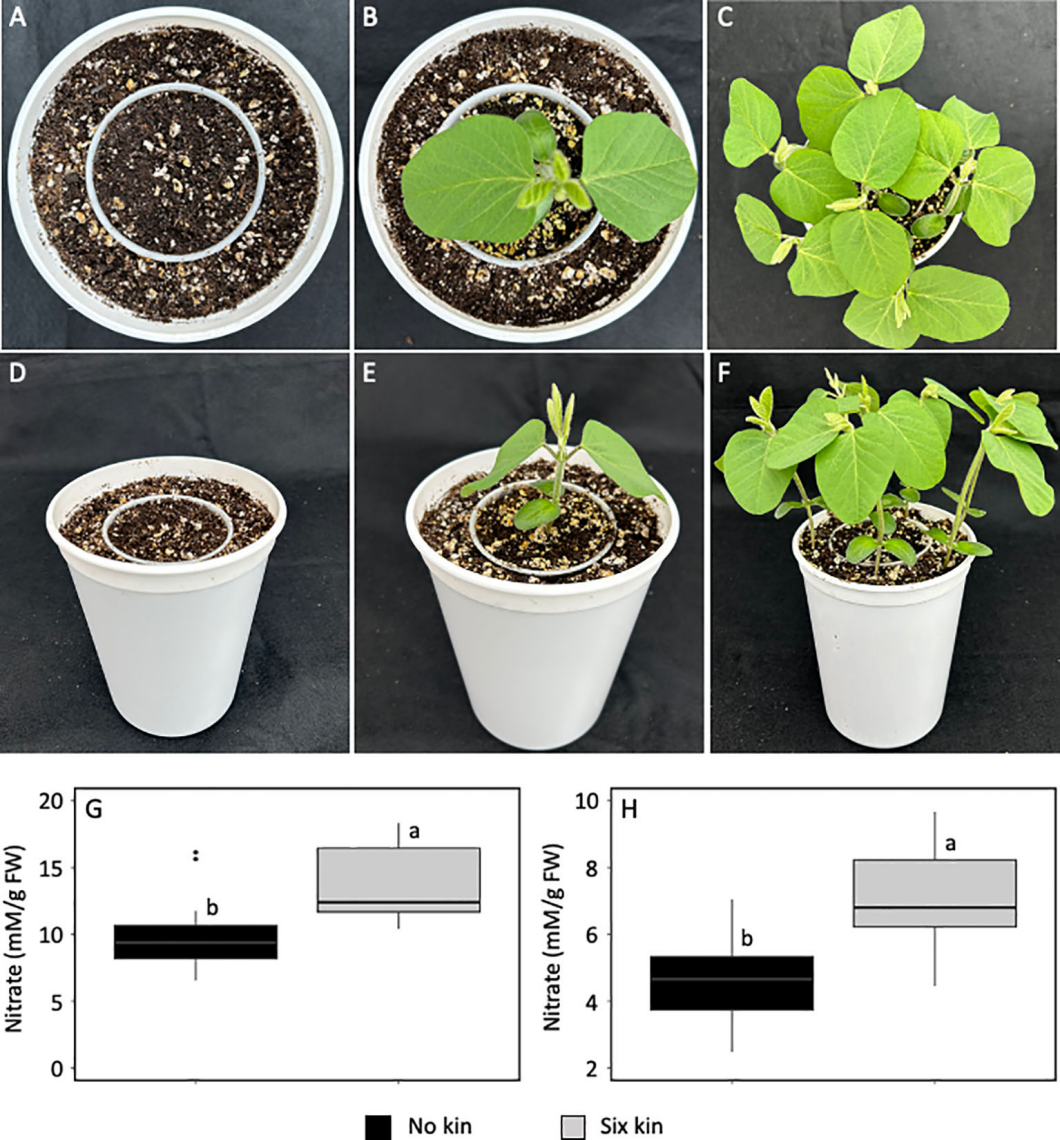
Experimental set up for intra-specific competition **(A)** and a soybean seedling (11-day-old) in the absence of kin seedlings **(B)** as a no kin control and in the presence of six kin seedlings **(C)** with R:FR ≈ 2.8 and ≈ 0.5, respectively. Plastic cups were placed in the center of the pots such that soil level in each plastic cup was matched with that in the surrounding area in the pot **(D)**. Normal growth of a soybean seedling in the absence of kin **(E)** and elongation growth in the presence of six kin seedlings **(F)**. Note that the plastic cup prevents direct roots contact between the soybean seedling in the cup and six surrounding kin seedlings in the pot. Increase in leaf nitrate level in the unifoliate leaves **(G)** and first trifoliate leaves **(H)** under intra-specific competition with six kin seedlings. The data are presented as least square means ± SE from three independent experiments, each with six plants per treatment. Means were compared using Tukey’s HSD test (*p*<0.05). Letters denote statistically significant differences among treatments.

Neighbouring kin plants that are not overlapping can generate low R:FR environments during intra-specific competition (Huber et al., 2021). We used soybean as a representative broad leaf plant to investigate whether the low R:FR light environment generated by kin plants during intra-specific competition can also increase leaf nitrate level (**Figures 3A-F**). We found that nitrate levels increased by 37% in the unifoliate leaves (**Figure 3G**) and 62% in the first trifoliate leaves (*p*<0.05) (**Figure 3H**), when individual soybean seedlings were raised along with six surrounding kin seedlings in the absence of direct root contact. These findings suggest that decreases in R:FR light by kin plants under higher crop plant densities may trigger nitrate accumulation during intra-specific competition.

### Increased nitrate concentration in maize leaves under low R:FR environments does not coincide with the upregulation of vacuolar nitrate transporter genes

We performed RNA-sequencing to gain insights into the impact of low R:FR light treatments on the expression of genes involved in nitrogen metabolism in maize leaves. In addition, given the nitrate accumulation in maize leaves and the crucial role of vacuolar nitrate transporter genes such as *CLCa* in nitrate storage in the vacuoles (Hodin et al., 2023), we sought to determine whether the low R:FR treatments had an effect on the expression of *CLCa* in maize leaves based on *CLCa* homologs in *Arabidopsis*.

As shown in **Table 1**, our data indicated that ≈ 90% of sequence reads were mapped to the maize reference genome. Further, 50.6% and 70.4% of DEGs were found to be specific to biological low R:FR light and artificial low R:FR light, respectively. On the other hand, 49.4% of DEGs in the biological low R:FR light treatment and 29.6% of DEGs in the artificial low R:FR light treatment were found to be common between the two treatments (**Table 2**).

**Table 1:** Percentages of paired-end sequence reads mapped to the maize reference genome.

Treatment	Replicate	No. base pairs sequenced	No. mapped reads	Percent mapped
Weed-free control	1	27248251	30306987	89.8%
	2	28428221	31768245	89.9%
	3	34095641	37835507	90.1%
Biological low R:FR	1	21443086	23940714	89.6%
	2	25824560	28984602	89.1%
	3	36645896	40657651	90.1%
Artificial low R:FR	1	30428000	33831687	89.9%
	2	20844175	23233666	89.7%
	3	27370162	30456614	89.9%
Average		28036443	31223963	89.7%

**Table 2:** Numbers and percentages of treatment-specific and common DEGs in the biological and artificial low R:FR light treatments relative to the control.

DEGs	Biological low R:FR	Percentage	Artificial low R:FR	Percentage
Specific	1153	50.6%	2676	70.4%
Common	1124	49.4%	1124	29.6%
Total	2277		3800	

Changes in the expression of genes encoding nitrate assimilation enzymes were not consistent in the biological and artificial low R:FR light treatments (**Table 3** and **Supplementary Table 2**). For example, down-regulation of an *NR* gene (Zm00001eb122960) and upregulation of a *GS* gene (Zm00001eb399860), and a *GOGAT* gene (Zm00001eb156610) occurred in the biological low R:FR light treatment only. On the other hand, two *NR* genes (Zm00001eb122720 and Zm00001eb193390) and two *GS* genes (Zm00001eb009090 and Zm00001eb054990) were expressed in opposite directions in the artificial low R:FR light treatment only (**Table 3** and **Supplementary Table 2**).

**Table 3:** Numbers of treatment-specific and common DEGs in the biological and artificial low R:FR light treatments involved in maize nitrogen metabolism.

Gene function	Biological low R:FR	Artificial low R:FR	Common DEGs	Total DEGs
Nitrate assimilation enzymes	3	4	0	7
	2 (+)	2 (+)	0 (+)	4 (+)
	1 (-)	2 (-)	0 (-)	3 (-)
Nitrate signaling	0	1	0	1
	0 (+)	0 (+)	0 (+)	0 (+)
	0 (-)	1 (-)	0 (-)	1 (-)
Nitrate transporters	1	1	0	2
	0 (+)	1 (+)	0 (+)	1 (+)
	1 (-)	0 (-)	0 (-)	1 (-)
Ammonium transporters	0	1	1	2
	0 (+)	0 (+)	0 (+)	0 (+)
	0 (-)	1 (-)	1 (-)	2 (-)
Peptide transporters	2	5	4	11
	2 (+)	2 (+)	1 (+)	5 (+)
	0 (-)	3 (-)	3 (-)	6 (-)
Amino acid transporters	2	8	3	13
	1 (+)	1 (+)	2 (+)	4 (+)
	1 (-)	7 (-)	1 (-)	9 (-)

(+): Upregulation; (-): Downregulation.

With regard to nitrate signaling, we found down-regulation of an *Arabidopsis* homolog of nitrate responsive NIN (nodule inception)-like transcription factor 6 (Zm00001eb339390) in the artificial low R:FR light treatment only, which is thought to be responsible for the expression of nitrate inducible genes in *Arabidopsis* (Konishi and Yanagisawa, 2013). Interestingly, no DEGs involved in nitrate signaling were found in the biological low R:FR light treatment (**Table 3** and **Supplementary Table 2**).

Given the importance of nitrate transporters in the uptake, transport, and storage of nitrate within plant tissue, we looked for DEGs encoding nitrate transporters in the biological and artificial low R:FR light treatment. We found an inconsistent upregulation of a nitrate transporter (Zm00001eb291130) with similarity to *Arabidopsis* NPF4.3 with unknown substrate (Léran et al., 2014) in the artificial low R:FR light, and downregulation of a nitrate transporter (Zm00001eb162310) with similarity to the fungal nitrate transporter *crnA* (*colonial restriction nitrate*) (Unkles et al., 1991) in the biological low R:FR light treatment (**Table 3** and **Supplementary Table 2**). In contrast, while an ammonium transporter (Zm00001eb063910) was consistently downregulated in the biological and artificial low R:FR light treatments, a second ammonium transporter (Zm00001eb247430) was downregulated in the artificial low R:FR treatment only (**Tables 3**, **4**, and **Supplementary Table 2**).

**Table 4:** Commonly up (+) or down (-) regulated ammonium, peptide, and amino acid transporter genes in the biological and artificial low R:FR light treatments.

Function	Biological low R:FR		Artificial low R:FR	
DEGs	Log_2_FC	*p*-value	Log_2_FC	*p*-value
Ammonium transporters				
Zm00001eb063910	-0.11	1.46E-03	-2.18	8.94E-05
Peptide transporters				
Zm00001eb025880	+0.57	5.53E-05	+0.75	2.02E-07
Zm00001eb287980	-0.66	1.42E-03	-0.40	1.30E-02
Zm00001eb245250	-0.65	4.09E-06	-0.66	7.49E-07
Zm00001eb371840	-0.95	6.5E-10	-1.24	7.32E-16
Amino acid transporters				
Zm00001eb080770	+1.49	1.46E-08	+1.14	2.59E-05
Zm00001eb303770	+0.66	2.88E-05	+0.51	7.70E-04
Zm00001eb349050	-0.71	1.57E-07	-0.53	7.21E-06

Increase in expression of peptide transporters can result in enhanced leaf nitrogen content and plant growth (Komarova et al., 2008). In maize leaves, we found that nine DEGs in the artificial low R:FR light treatment and six DEGs in the biological low R:FR light treatment encoded peptide transporters (**Tables 3**, **4** and **Supplementary Table 2**). The upregulation of one DEG (Zm00001eb025880) and downregulation of three DEGs (Zm00001eb287980, Zm00001eb245250, and Zm00001eb371840) commonly occurred in both treatments (**Tables 3**, **4** and **Supplementary Table 2**). The proteins encoded by Zm00001eb025880 and Zm00001eb287980 displayed highest similarities (84%, and 87%) to orthologs of unknown function in *Sorghum bicolor* (L.) Moench (SORBI 3001G276400 and SORBI 3009G136700, respectively). The protein encoded by Zm00001eb245250, however, showed even greater similarity (89%) to an ortholog in *Sorghum bicolor* (SORBI 3004G193000) and to a lesser extent (61%) to an ortholog in *Arabidopsis* (AT3G21670). These two orthologs encode a member of the NRT1/PTR (nitrate transporter/peptide transporter) family 6.4, where most of the members are known to be nitrate transporters (Leran et al., 2014). The encoded protein by Zm00001eb371840 showed highest identity (84%) to an ortholog in *Sorghum bicolor* (SORBI 3010G099700), which encodes a putative oligopeptide transporter. It shows, however, a low identity (43%) to an ortholog in *Arabidopsis* (AT5G14940), which has been identified (NPF5.8) as an exporter of the metal chelator nicotianamine (Chao et al., 2021).

Since amino acid transporters can also influence nitrate levels in plant leaves (Perchlik and Tegeder, 2018), we looked for DEGs encoding amino acid transporters in maize leaves in the biological and artificial low R:FR light treatments. We found that 11 DEGs in the artificial low R:FR light treatment and five DEGs in the biological low R:FR light treatment encoded amino acid transporters. Among these, only the upregulation Zm00001eb080770 and Zm00001eb303770, and downregulation of Zm00001eb349050 in the biological low R:FR light treatment, were consistent with those in the artificial low R:FR light treatment and therefore, these transcriptional changes were most likely due to the reflected FR light from neighbouring weeds (**Tables 3**, **4** and **Supplementary Table 2**). The protein encoded by Zm00001eb080770 is predicted to be an amino acid/auxin permease11, which displays 75% and 83% identity to it orthologs in monocot grass species *Brachypodium distachyon* (L.) P.Beauv. and *Setaria italica* (L.) P.Beauv., respectively. These two orthologs, however, are hypothetical proteins of unknown function. The encoded protein by Zm00001eb303770 is highly identical (93%) to its ortholog of unknown function in *Sorghum bicolor* (SORBI 3002G082900) but displays low identity (44%) to its ortholog the *Arabidopsis nitrate transporter 1* (*ANT1*) (AT3G11900), which encodes an aromatic and neutral amino acid transporter involved in moving amino acids out of the phloem (Yao et al., 2020) and its expression is known to be regulated by nitrate (Liu and Bush, 2006).

Overall, the possibility that the above transcriptional responses to low R:FR light may influence nitrate content of maize leaves cannot be excluded. While the RNA-seq results revealed a range of differentially expressed genes that are proven to be linked to nitrate metabolism and transport, we did not observe evidence specifically implicating vacuolar nitrate transporter genes in the regulation of nitrate levels under low R:FR light conditions.

### Increase in nitrate concentration of maize leaves under low R:FR environments coincides with a decrease in fd-GOGAT activity

**Figure 4:**
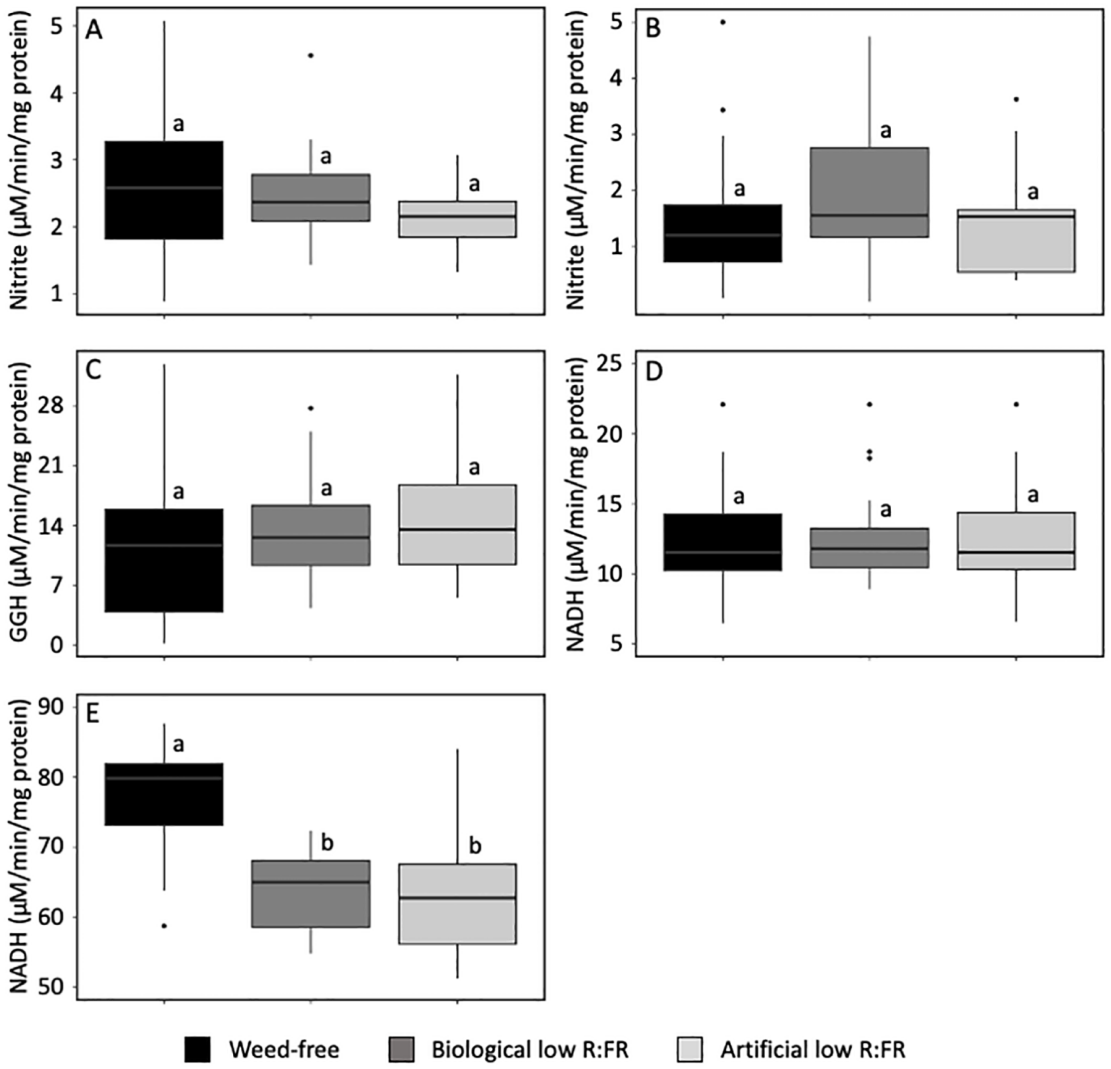
Activities of nitrate reductase, NR **(A)** nitrite reductase, NiR **(B)** glutamine synthetase, GS **(C)** NADH-dependent glutamine:2-oxoglutarate aminotransferase, NADH-GOGAT **(D)** and ferredoxin-dependent glutamine:2-oxoglutarate aminotransferase, fd-GOGAT **(E)** in the second leaf of maize seedlings (9-day-old) in the weed-free control, biological low R:FR light, and artificial low R:FR light treatments with R:FR ≈ 2.8, ≈ 0.5, and ≈ 0.5, respectively. fd, ferredoxin; GGH, gamma glutamyl hydroxamate; NADH, beta-nicotinamide adenine dinucleotide. Note that the low R:FR light treatments changed the fd-GOGAT activity only. The data are presented as least square means ± SE from three independent experiments, each with six plants per treatment. Means were compared using Tukey’s HSD test (*p*<0.05). Letters denote statistically significant differences among treatments.

In higher plants, light plays important roles in the regulation of activities of nitrate assimilation enzymes (Fan et al., 2019; Suzuki et al., 2001; Teller et al., 1996; Elmlinger and Mohr, 1991; Hecht et al., 1988). To gain insights into the impact of the low R:FR treatments on the activities of nitrate assimilation enzymes in maize laves and a possible relationship between these activities and nitrate accumulation, we compared the activities of NR, NiR, GS, NADH-GOGAT, and fd-GOGAT in maize leaves in the low R:FR light treatments with that in the weed-free control. The biological and artificial low R:FR light treatments did not result in significant changes in the activities of NR, NiR, GS, and NADH-GOGAT compared with that in the control (**Figures 4A-D**). In contrast, the biological and artificial low R:FR light treatments resulted in 30% and 29% decrease in fd-GOGAT activity, respectively (*p*<0.05) (**Figure 4E**). This decrease in activity of a single enzyme throughout the nitrate assimilation pathway may be a contributing factor to increases in nitrate concentration in maize leaves in the biological and artificial low R:FR light treatments and further suggests the specificity of the low R:FR action on the fd-GOGAT activity in maize leaves during weed competition.

### Thioredoxins may play a role in modulation of nitrate metabolism under low R:FR environments

We found 15 DEGs in the biological low R:FR light treatment and 11 DEGs in the artificial low R:FR light treatment that encoded thioredoxins (TRXs). Analysis of subcellular localizations of these TRXs by the protein subcellular localization prediction tool WolF PSORT (https://wolfpsort.hgc.jp) predicted that nine (out of 15) TRXs in the biological low R:FR light treatment and eight (out of 11) TRXs in the artificial low R:FR light treatment were localized in the chloroplast. Among these, three chloroplast-localized TRXs displayed similar expression patterns in both treatments (**Table 5**, **Supplementary Table 2**). It is known that reduced ferredoxin transfers electrons to TRXs via the action of ferredoxin thioredoxin reductase, which enables TRXs to reduce disulfide bonds on their target enzymes (Lemaire et al., 2007). In this regard, four DEGs in the biological low R:FR light treatment and six DEGs in the artificial low R:FR light treatment were found to encode ferredoxin. Among these, three DEGs displayed similar expression patterns in both treatments (**Table 5**, **Supplementary Table 2**). Although TRXs regulate multitude of chloroplast processes (Lemaire et al., 2007), these transcriptional responses raise the possibility of involvement of TRXs in regulation of nitrate metabolism under low R:FR environments.

**Table 5:** Predicted subcellular localization of commonly up (+) or down (-) regulated thioredoxin and ferredoxin genes in the biological and artificial low R:FR light treatments.

Function	Biological low R:FR		Artificial low R:FR		WoLF PSORT
DEGs	Log_2_FC	*p*-value	Log_2_FC	*p*-value	prediction
Thioredoxin genes					
Zm00001eb193260	+0.46	6.84E-04	+0.97	2.17E-10	Chloroplast
Zm00001eb042600	+0.59	2.77E-06	+0.69	2.64E-07	Chloroplast
Zm00001eb015550	-0.26	5.77E-03	-0.60	3.34E-05	Chloroplast
Ferredoxin genes					
Zm00001eb421930	+0.93	1.23E-03	+1.38	3.03E-05	Chloroplast
Zm00001eb083950	+0.64	5.56E-05	+0.69	3.63E-05	Chloroplast
Zm00001eb174420	-0.92	2.71E-09	-0.30	4.22E-03	Chloroplast

## Discussion

Activation of SAS (Ballaré and Pierik, 2017) is an adaptive response to low R:FR light signals generated by proximate vegetation (Schmitt et al., 2003). Although this adaptive response supports plant survival against competitors, it can adversely affect crop fitness, disease resistance, and yield potential (Evers et al., 2006; Warnasooriya and Brutnell, 2014; Libenson et al., 2002; Ballaré, 2014; Boccalandro et al., 2003; Weijschedé et al., 2006; Courbier et al., 2021; De Wit et al., 2013; Xiang et al., 2021; Xie et al., 2011). These adverse effects are caused by a myriad of changes at the subcellular to whole plant level. The present study provides evidence that low R:FR signals can also cause changes in the nitrate assimilation pathway. The increase in leaf nitrate concentration appears to be a specific response to the presence of neighbouring weeds as we found it to occur in corn and soybean. The demonstration of a similar response to artificial low R:FR light strongly suggests that nitrate accumulation in the biological low R:FR light treatment is due to reflected FR light from neighbouring weeds and it may be a component of the low R:FR-induced stress response (**Figure 1** and **2**). Further, a similar increase in leaf nitrate concentration in individual soybean seedlings grown simultaneously with six neighbouring kin seedlings (**Figure 3**) suggests the possibility of nitrate accumulation in response to the low R:FR signals generated during intra-specific competition that occurs under high density planting.

Decreased leaf fd-GOGAT activity in the biological and artificial low R:FR light treatments appeared to be a unique response to reflected FR light as activities of other nitrate assimilation enzymes remained unchanged (**Figure 4**). This finding is consistent with a previous report, where fd-GOGAT activity in etiolated maize leaves was increased by red light pulses and reversibly repressed by far-red light pulses while NADH-GOGAT activity remained constant (Suzuki et al., 2001). In addition, phytochrome-mediated regulation of fd-GOGAT activity has been demonstrated in the turions of *Spirodela polyrhiza*, where red light-mediated activation of fd-GOGAT was reverted by far-red light (Teller et al., 1996). The GS/fd-GOGAT cycle in the leaf chloroplasts is the main pathway for primary nitrogen assimilation and photorespiratory ammonium re-assimilation (Potel et al., 2009; Coschigano et al., 1998). A dramatic decrease in fd-GOGAT activity in the rice *ABC1-1* (*ABNORMAL CYTOKININ RESPONSE 1*) mutant was accompanied by increases in soluble sugar and total nitrogen levels (Yang et al., 2016). We also found that the low R:FR-mediated decrease in leaf fd-GOGAT activity coincided with increased nitrate concentration in maize leaves (**Figure 1** and **4**). Decreased fd-GOGAT activity may not be the sole factor responsible for the low R:FR light-mediated nitrate accumulation in maize leaves. The involvement of other factors such as increased root to shoot translocation of nitrate and its storage in the vacuole cannot be excluded. The importance of vacuoles as a major nitrate storage pool was highlighted in earlier studies, where 58% and 99% of barley protoplast nitrate were found in the vacuoles (Martinoia et al., 1981; Granstedt and Huffaker, 1982). As shown previously in barley leaves, increased nitrate influx to the small ‘metabolic’ pool would induce NR activity, while diversion of nitrate away from the site of metabolism and its transfer to the vacuole as a large ‘storage’ pool resulted in hardly detectable NR activity in the vacuolar preparations (Martinoia et al., 1981). In a similar manner, increased leaf nitrate concentration and the lack of induction of NR activity in the low R:FR light treatments may be indicative of nitrate accumulation in the vacuoles.

It is known that nitrate plays a role as an osmoticum in the vacuole compensating for soluble carbohydrates and organic acids, whose synthesis decline under light limiting conditions (Umar and Iqbal, 2007). We surmised that nitrate accumulation in maize leaves might have occurred in the vacuole in exchange for soluble carbohydrates required for rapid elongation growth under low R:FR light. Such replacement of soluble carbohydrates with nitrate may require changes in the expression of vacuolar nitrate transporters. Vacuolar nitrate storage and efflux are regulated by an array of vacuolar nitrate transporters (De Angeli et al., 2006; Lu et al., 2022). For example, *Arabidopsis* CLCa is a nitrate proton antiporter that is localized in the tonoplast and transports nitrate from the cytosol to the vacuolar lumen (De Angeli et al., 2006). Further, disruption of *AtCLC-a* by T-DNA insertion resulted in a dramatic reduction in nitrate content under excess nitrate suggesting a specific role for AtCLC-a in modulating nitrate status (Geelen et al., 2000). In contrast to our assumption, no orthologs of *Arabidopsis* vacuolar nitrate transporters were found among DEGs in the low R:FR treatments (**Supplementary Table 2**). In addition, two DEGs encoding nitrate transporters displayed opposing responses in the artificial and biological low R:FR treatments (**Supplementary Table 2**). Overall, our data (**Tables 3**, **4**, and **Supplementary Table 2**) indicate that both the biological and artificial low R:FR light treatments influence the expression of transporter genes involved in the transport of nitrate, ammonium, peptides, and amino acids, however, the magnitude and direction of regulation vary across different environments. The artificial low R:FR light treatment appeared to have a more significant effect on the expression of ammonium transporters. Peptide transporters were predominantly downregulated, again, with stronger effects in the artificial low R:FR light treatment. Meanwhile, amino acid transporter genes showed a more varied response, with both upregulation and downregulation observed depending on the specific gene and treatment. These findings highlight the complexity of maize responses to light quality and further suggest that changes in R:FR light under biological conditions may have a dramatically different impact on transcription of certain transporter genes compared with that under artificially manipulated R:FR light conditions. Given the important roles of nitrate, peptide, and amino acid transporters in the regulation of nitrate levels within plants, we do not rule out the possibility that the above changes in the expression of nitrate, peptide, and amino acid transporters may contribute to nitrate accumulation in maize leaves in the low R:FR light treatments.

Thioredoxins (TRXs) play major roles in the regulation of chloroplast processes including nitrogen metabolism (Lemaire et al., 2007). In particular, NiR (Marchand et al., 2004), GS (Motohashi et al., 2001; Balmer et al., 2004), and fd-GOGAT (Lichter and Haüberlein, 1998) are known TRX targets. The concurrent transcriptional responses of TRXs and ferredoxins suggest that the TRXs, which are commonly expressed in the biological and artificial low R:FR light treatments (**Table 5**), may be involved in the regulation of chloroplast processes including nitrate metabolism in response to weed competition cues.

Several nitrate transporters are involved in nitrate influx into roots and in root xylem loading that distribute nitrate to the shoot (Krapp et al., 2014). We did not assess the transcript levels of nitrate transporters in roots as manipulation of root samples including soil removal or root washing and drying could affect root transcriptome responses thus confounding the low R:FR-specific effects. Therefore, our experiments cannot rule out the possibility that a low R:FR light-mediated induction of root nitrate transporters may increase nitrate transport from roots to shoots. We are, however, not aware of any previous study that provides a link between a shoot perceived low R:FR signal and induction of a root nitrate transporter gene. Further, a brief exposure to FR light did not affect nitrate uptake by etiolated rice seedlings suggesting the lack of involvement of phytochrome in nitrate uptake (Sasakawa and Yamamoto, 1979). It is, however, worthwhile mentioning that repression of *Arabidopsis* lateral root development by a shoot perceived low R:FR signal occurs via the transport of HY5 to the root and indirect repression of NRT2.1 (Van Gelderen et al., 2021).

In conclusion, perception of low R:FR signals generated by neighbouring weeds leads to accumulation of nitrate in maize leaves. The occurrence of a similar response in distantly related species including soybean suggests that the low R:FR-mediated accumulation of nitrate may be an important component of SAS. Although a multitude of factors may be involved in nitrate accumulation in plant leaves, a reduction in fd-GOGAT activity in maize leaves appears to be a contributing factor to the low R:FR light-mediated nitrate accumulation. Given the established action of nitrate accumulation in the plant shoot as a signal to regulate biomass partitioning between shoot and root, the low R:FR light-mediated accumulation of nitrate in leaves may act as a signal that causes typical decreases in root to shoot ratios in crop plants under weed competition. Lastly, our findings are not intended to serve as predictor of nitrate accumulation at the whole plant level as the plants mature under competition stress but rather a snapshot of time, when the seedling response to low R:FR is readily detectable. These findings warrant further research into the duration of nitrate accumulation and the time of recovery following the removal of competition stress in agricultural settings.

## Data Availability

The datasets presented in this study can be found in online repositories. The names of the repository/repositories and accession number(s) can be found in the article/**Supplementary Material**.

## References

[B1] Ali A. Sivakami S. Raghuram N. (2007). Effect of nitrate, nitrite, ammonium, glutamate, glutamine and 2-oxoglutarate on the RNA levels and enzyme activities nitrate reductase and nitrite reductase in rice. Physiol. Mol. Biol. Plants 13, 17–25.

[B2] Ballaré C. L. (2014). Light regulation of plant defense. Annu. Rev. Plant Biol. 65, 335–363. doi: 10.1146/annurev-arplant-050213-040145, PMID: 24471835

[B3] Ballaré C. L. Pierik R. (2017). The shade-avoidance syndrome: multiple signals and ecological consequences. Plant Cell Environ. 40, 2530–2543. doi: 10.1111/pce.12914, PMID: 28102548

[B4] Balmer Y. Koller A. Val G. D. Schürmann P. Buchanan B. B. (2004). Proteomics uncovers proteins interacting electrostatically with thioredoxin in chloroplasts. Photosynth. Res. 79, 275–280. doi: 10.1023/B:PRES.0000017207.88257.d4, PMID: 16328793

[B5] Beevers L. Schrader L. E. Flesher D. Hageman R. H. (1965). The Role of light and nitrate in the induction of nitrate reductase in radish cotyledons and maize seedlings. Plant Physiol. 40, 691–698. doi: 10.1104/pp.40.4.691, PMID: 16656145 PMC550363

[B6] Bian Z. Wang Y. Zhang X. Li T. Grundy S. Yang Q.. (2020). A review of environment effects on nitrate accumulation in leafy vegetables grown in controlled environments. Foods (Basel Switzerland) 9, 732. doi: 10.3390/foods9060732, PMID: 32503134 PMC7353485

[B7] Boccalandro H. E. Ploschuk E. L. Yanovsky M. J. Sánchez R. A. Gatz C. Casal J. J. (2003). Increased phytochrome B alleviates density effects on tuber yield of field potato crops. Plant Physiol. 133, 1539–1546. doi: 10.1104/pp.103.029579, PMID: 14605224 PMC300711

[B8] Bräutigam A. Gagneul D. Weber A. P. (2007). High-throughput colorimetric method for the parallel assay of glyoxylic acid and ammonium in a single extract. Anal. Biochem. 362, 151–153. doi: 10.1016/j.ab.2006.12.033, PMID: 17222384

[B9] Brunswick P. Cresswell C. F. (1988). Nitrite uptake into intact pea chloroplasts: I. Kinetics and relationship with nitrite assimilation. Plant Physiol. 86, 378–383. doi: 10.1104/pp.86.2.378, PMID: 16665916 PMC1054492

[B10] Cataldo D. A. Maroon M. Schrader L. E. Youngs V. L. (1975). Rapid colorimetric determination of nitrate in plant tissue by nitration of salicylic acid. Commun. Soil Sci. Plant Anal. 6, 71–80. doi: 10.1080/00103627509366547

[B11] Chao Z. F. Wang Y. L. Chen Y. Y. Zhang C. Y. Wang P. Y. Song T.. (2021). NPF transporters in synaptic-like vesicles control delivery of iron and copper to seeds. Sci. Adv. 7, eabh2450. doi: 10.1126/sciadv.abh2450, PMID: 34516912 PMC8442890

[B12] Chen S. Zhou Y. Chen Y. Gu J. (2018). fastp: an ultra-fast all-in-one FASTQ preprocessor. Bioinformatics 34, i884–i890. doi: 10.1093/bioinformatics/bts635, PMID: 30423086 PMC6129281

[B13] Coschigano K. T. Melo-Oliveira R. Lim J. Coruzzi G. M. (1998). *Arabidopsis gls* mutants and distinct Fd-GOGAT genes. Implications for photorespiration and primary nitrogen assimilation. Plant Cell 10, 741–752. doi: 10.1105/tpc.10.5.741, PMID: 9596633 PMC144371

[B14] Courbier S. Snoek B. L. Kajala K. Li L. van Wees S. C. M. Pierik R. (2021). Mechanisms of far-red light-mediated dampening of defense against *Botrytis cinerea* in tomato leaves. Plant Physiol. 187, 1250–1266. doi: 10.1093/plphys/kiab354, PMID: 34618050 PMC8566310

[B15] De Angeli A. Monachello D. Ephritikhine G. Frachisse J. M. Thomine S. Gambale F.. (2006). The nitrate/proton antiporter AtCLCa mediates nitrate accumulation in plant vacuoles. Nature 442, 939–942. doi: 10.1038/nature05013, PMID: 16878138

[B16] Dechorgnat J. Nguyen C. T. Armengaud P. Jossier M. Diatloff E. Filleur S.. (2011). From the soil to the seeds: the long journey of nitrate in plants. J. Exp. Bot. 62, 1349–1359. doi: 10.1093/jxb/erq409, PMID: 21193579

[B17] De Wit M. Spoel S. H. Sanchez-Perez G. F. Gommers C. M. M. Pieterse C. M. J. Voesenek L. A. C. J.. (2013). Perception of low red:far-red ratio compromises both salicylic acid- and jasmonic acid-dependent pathogen defences in *Arabidopsis* . Plant J. 75, 90–103. doi: 10.1111/tpj.12203, PMID: 23578319

[B18] Dobin A. Davis C. A. Schlesinger F. Drenkow J. Zaleski C. Jha S.. (2013). STAR: ultrafast universal RNA-seq aligner. Bioinformatics 29, 15–21. doi: 10.1093/bioinformatics/bts635, PMID: 23104886 PMC3530905

[B19] Elmlinger M. W. Mohr H. (1991). Coaction of blue/ultraviolet-A light and light absorbed by phytochrome in controlling the appearance of ferredoxin-dependent glutamate synthase in the Scots pine (*Pinus sylvestris* L.) seedling. Planta 183, 374–380. doi: 10.1007/BF00197736, PMID: 24193748

[B20] Esposito S. Guerriero G. Vona V. Di Martino Rigano V. Carfagna S. Rigano C. (2005). Glutamate synthase activities and protein changes in relation to nitrogen nutrition in barley: the dependence on different plastidic glucose-6P dehydrogenase isoforms. J. Exp. Bot. 56, 55–64. doi: 10.1093/jxb/eri006, PMID: 15501908

[B21] Evers J. B. Vos J. Andrieu B. Struik P. C. (2006). Cessation of tillering in spring wheat in relation to light interception and red: far-red ratio. Ann. Bot. 97, 649–658. doi: 10.1093/aob/mcl020, PMID: 16464875 PMC2803660

[B22] Fan S. C. Lin C. S. Hsu P. K. Lin S. H. Tsay Y. F. (2009). The *Arabidopsis* nitrate transporter NRT1.7, expressed in phloem, is responsible for source-to-sink remobilization of nitrate. Plant Cell 21, 2750–2761. doi: 10.1105/tpc.109.067603, PMID: 19734434 PMC2768937

[B23] Fan X. Xue F. Song B. Chen L. Xu G. Xu H. (2019). Effects of blue and red light on growth and nitrate metabolism in pakchoi. Open Chem. 17, 456–464. doi: 10.1515/chem-2019-0038

[B24] Geelen D. Lurin C. Bouchez D. Frachisse J. M. Lelièvre F. Courtial B.. (2000). Disruption of putative anion channel gene *AtCLC-a* in *Arabidopsis* suggests a role in the regulation of nitrate content. Plant J. 21, 259–267. doi: 10.1046/j.1365-313x.2000.00680.x, PMID: 10758477

[B25] Granstedt R. C. Huffaker R. C. (1982). Identification of the leaf vacuole as a major nitrate storage pool. Plant Physiol. 70, 410–413. doi: 10.1104/pp.70.2.410, PMID: 16662506 PMC1067160

[B26] Hecht U. Oelmüller R. Schmidt S. Mohr H. (1988). Action of light, nitrate and ammonium on the levels of NADH- and ferredoxin-dependent glutamate synthases in the cotyledons of mustard seedlings. Planta 175, 130–138. doi: 10.1007/BF00402890, PMID: 24221637

[B27] Hoagland D. R. (1933). The water-culture method for growing plants without soil. Calif. Agric. Exp. Stn. Circ. 347, 1–39.

[B28] Hodin J. Lind C. Marmagne A. Espagne C. Bianchi M. W. De Angeli A.. (2023). Proton exchange by the vacuolar nitrate transporter CLCa is required for plant growth and nitrogen use efficiency. Plant Cell 35, 318–335. doi: 10.1093/plcell/koac325, PMID: 36409008 PMC9806559

[B29] Hu Y. Fernández V. Ma L. (2014). Nitrate transporters in leaves and their potential roles in foliar uptake of nitrogen dioxide. Front. Plant Sci. 5. doi: 10.3389/fpls.2014.00360, PMID: 25126090 PMC4115617

[B30] Huang L. Zhang H. Zhang H. Deng X. W. Wei N. (2015). *HY5* regulates nitrite reductase 1 (*NIR1*) and ammonium transporter1;2 (*AMT1;2*) in *Arabidopsis* seedlings. Plant Sci. 238, 330–339. doi: 10.1016/j.plantsci.2015.05.004, PMID: 26259199 PMC4719586

[B31] Huber M. Nieuwendijk N. M. Pantazopoulou C. K. Pierik R. (2021). Light signalling shapes plant-plant interactions in dense canopies. Plant Cell Environ. 44, 1014–1029. doi: 10.1111/pce.13912, PMID: 33047350 PMC8049026

[B32] Johnson C. F. Langhans R. W. Albright L. D. Combs G. F. Welch R. M. Heller L.. (1999). Spinach: Nitrate analysis of an advanced life support (ALS) crop cultured under ALS candidate artificial light sources. SAE Tech. Paper, 1999–01-2107. doi: 10.4271/1999-01-2107

[B33] Jonassen E. M. Lea U. S. Lillo C. (2008). *HY5* and *HYH* are positive regulators of nitrate reductase in seedlings and rosette stage plants. Planta 227, 559–564. doi: 10.1007/s00425-007-0638-4, PMID: 17929051

[B34] Jones R. W. Sheard R. W. (1972). Nitrate reductase activity: phytochrome mediation of induction in etiolated peas. Nature 238, 221–222. doi: 10.1038/newbio238221a0, PMID: 4506207

[B35] Knezevic S. Z. Datta A. (2015). The critical period for weed control: Revisiting data analysis. Weed Sci. 63, 188–202. doi: 10.1614/WS-D-14-00035.1

[B36] Komarova N. Y. Thor K. Gubler A. Meier S. Dietrich D. Weichert A.. (2008). AtPTR1 and AtPTR5 transport dipeptides in planta. Plant Physiol. 148, 856–869. doi: 10.1104/pp.108.123844, PMID: 18753286 PMC2556804

[B37] Konishi M. Yanagisawa S. (2013). *Arabidopsis* NIN-like transcription factors have a central role in nitrate signalling. Nat. Commun. 4, 1617. doi: 10.1038/ncomms2621, PMID: 23511481

[B38] Krapp A. (2015). Plant nitrogen assimilation and its regulation: a complex puzzle with missing pieces. Curr. Opin. Plant Biol. 25, 115–122. doi: 10.1016/j.pbi.2015.05.010, PMID: 26037390

[B39] Krapp A. David L. C. Chardin C. Girin T. Marmagne A. Leprince A. S.. (2014). Nitrate transport and signalling in *Arabidopsis* . J. Exp. Bot. 65, 789–798. doi: 10.1093/jxb/eru001, PMID: 24532451

[B40] Larson E. Howlett B. Jagendorf A. (1986). Artificial reductant enhancement of the Lowry method for protein determination. Anal. Biochem. 155, 243–248. doi: 10.1016/0003-2697(86)90432-x, PMID: 3728976

[B41] Lea P. J. Blackwell R. D. Chen F. L. Hetch U. (1990). “The enzymes of ammonia assimilation,” in Methods in plant biochemistry, vol. 3 . Ed. Lea P. J. (London: Acad. Press), 257–276.

[B42] Lemaire S. D. Michelet L. Zaffagnini M. Massot V. Issakidis-Bourguet E. (2007). Thioredoxins in chloroplasts. Curr. Genet. 51, 343–365. doi: 10.1007/s00294-007-0128-z, PMID: 17431629

[B43] Léran S. Varala K. Boyer J. C. Chiurazzi M. Crawford N. Daniel-Vedele F.. (2014). A unified nomenclature of NITRATE TRANSPORTER 1/PEPTIDE TRANSPORTER family members in plants. Trends Plant Sci. 19, 5–9. doi: 10.1016/j.tplants.2013.08.008, PMID: 24055139

[B44] Liao Y. Smyth G. K. Shi W. (2014). featureCounts: an efficient general purpose program for assigning sequence reads to genomic features. Bioinformatics 30, 923–930. doi: 10.1093/bioinformatics/btt656, PMID: 24227677

[B45] Libenson S. Rodriguez V. Pereira M. L. Sánchez R. A. Casal J. J. (2002). Low red to far-red ratios reaching the stem reduce grain yield in sunflower. Crop Sci. 42, 1180–1185. doi: 10.2135/cropsci2002.1180

[B46] Lichter A. Haüberlein I. (1998). A light-dependent redox signal participates in the regulation of ammonia fixation in chloroplasts of higher plants-ferredoxin:glutamate synthase is a thioredoxin-dependent enzyme. J. Plant Physiol. 153, 83–90. doi: 10.1016/S0176-1617(98)80049-7

[B47] Lillo C. (2008). Signalling cascades integrating light-enhanced nitrate metabolism. Biochem. J. 415, 11–19. doi: 10.1042/BJ20081115, PMID: 18778247

[B48] Lillo C. Appenroth K. J. (2001). Light regulation of nitrate reductase in higher plants: Which photoreceptors are involved? Plant Biol. 3, 455–465. doi: 10.1055/s-2001-17732

[B49] Liu X. Bush D. R. (2006). Expression and transcriptional regulation of amino acid transporters in plants. Amino Acids 30, 113–120. doi: 10.1007/s00726-005-0248-z, PMID: 16525755

[B50] Love M. I. Huber W. Anders S. (2014). Moderated estimation of fold change and dispersion for RNA-seq data with DESeq2. Genome Biol. 15, 550. doi: 10.1186/s13059-014-0550-8, PMID: 25516281 PMC4302049

[B51] Lu Y. T. Liu D. F. Wen T. T. Fang Z. J. Chen S. Y. Li H.. (2022). Vacuolar nitrate efflux requires multiple functional redundant nitrate transporter in *Arabidopsis thaliana* . Front. Plant Sci. 13. doi: 10.3389/fpls.2022.926809, PMID: 35937356 PMC9355642

[B52] Marchand C. Le Maréchal P. Meyer Y. Miginiac-Maslow M. Issakidis-Bourguet E. Decottignies P. (2004). New targets of *Arabidopsis* thioredoxins revealed by proteomic analysis. Proteomics 4, 2696–2706. doi: 10.1002/pmic.200400805, PMID: 15352244

[B53] Martinoia E. Heck U. Wiemken A. (1981). Vacuoles as storage compartments for nitrate in barley leaves. Nature 289, 292–294. doi: 10.1038/289292a0

[B54] Masclaux-Daubresse C. Daniel-Vedele F. Dechorgnat J. Chardon F. Gaufichon L. Suzuki A. (2010). Nitrogen uptake, assimilation and remobilization in plants: challenges for sustainable and productive agriculture. Ann. Bot. 105, 1141–1157. doi: 10.1093/aob/mcq028, PMID: 20299346 PMC2887065

[B55] Motohashi K. Kondoh A. Stumpp M. T. Hisabori T. (2001). Comprehensive survey of proteins targeted by chloroplast thioredoxin. Proc. Natl. Acad. Sci. U.S.A. 98, 11224–11229. doi: 10.1073/pnas.191282098, PMID: 11553771 PMC58711

[B56] Neininger A. Kronenberger J. Mohr H. (1992). Coaction of light, nitrate and a plastidic factor in controlling nitrite-reductase gene expression in tobacco. Planta 187, 381–387. doi: 10.1007/BF00195662, PMID: 24178079

[B57] Page E. R. Tollenaar M. Lee E. A. Lukens L. Swanton C. J. (2009). Does the shade avoidance response contribute to the critical period for weed control in maize (*Zea mays*)? Weed Res. 49, 563–571. doi: 10.1111/j.1365-3180.2009.00735.x

[B58] Pajuelo P. Pajuelo E. Forde B. G. Márquez A. J. (1997). Regulation of the expression of ferredoxin-glutamate synthase in barley. Planta 203, 517–525. doi: 10.1007/s004250050221, PMID: 9421934

[B59] Perchlik M. Tegeder M. (2018). Leaf amino acid supply affects photosynthetic and plant nitrogen use efficiency under nitrogen stress. Plant Physiol. 178, 174–188. doi: 10.1104/pp.18.00597, PMID: 30082496 PMC6130036

[B60] Pilgrim M. L. Caspar T. Quail P. H. McClung C. R. (1993). Circadian and light-regulated expression of nitrate reductase in *Arabidopsis* . Plant Mol. Biol. 23, 349–364. doi: 10.1007/BF00029010, PMID: 8219070

[B61] Potel F. Valadier M. H. Ferrario-Méry S. Grandjean O. Morin H. Gaufichon L.. (2009). Assimilation of excess ammonium into amino acids and nitrogen translocation in *Arabidopsis thaliana*- roles of glutamate synthases and carbamoylphosphate synthetase in leaves. FEBS J. 276, 4061–4076. doi: 10.1111/j.1742-4658.2009.07114.x, PMID: 19555410

[B62] Raghuram N. Sopory S. K. (1999). Roles of nitrate, nitrite and ammonium ion in phytochrome regulation of nitrate reductase gene expression in maize. Biochem. Mol. Biol. Int. 47, 239–249. doi: 10.1080/15216549900201253, PMID: 10205669

[B63] Rajasekhar V. K. Gowri G. Campbell W. H. (1988). Phytochrome-mediated light regulation of nitrate reductase expression in squash cotyledons. Plant Physiol. 88, 242–244. doi: 10.1104/pp.88.2.242, PMID: 16666287 PMC1055560

[B64] Ruberti I. Sessa G. Ciolfi A. Possenti M. Carabelli M. Morelli G. (2012). Plant adaptation to dynamically changing environment: the shade avoidance response. Biotechnol. Adv. 30, 1047–1058. doi: 10.1016/j.bioteChadv.2011.08.014, PMID: 21888962

[B65] Sakakibara H. Kawabata S. Hase T. Sugiyama T. (1992). Differential effect of nitrate and light on the expression of glutamine synthetase and ferredoxin-dependent glutamate synthase in maize. Plant Cell Physiol. 33, 1193–1198. doi: 10.1093/oxfordjournals.pcp.a078373

[B66] Sasakawa H. Yamamoto Y. (1979). Effects of red, far red, and blue light on enhancement of nitrate reductase activity and on nitrate uptake in etiolated rice seedlings. Plant Physiol. 63, 1098–1101. doi: 10.1104/pp.63.6.1098, PMID: 16660864 PMC542977

[B67] Schambow T. J. Adjesiwor A. T. Lorent L. Kniss A. R. (2019). Shade avoidance cues reduce *Beta vulgaris* growth. Weed Sci. 67, 311–317. doi: 10.1017/wsc.2019.2

[B68] Schmitt J. Stinchcombe J. R. Heschel M. S. Huber H. (2003). The adaptive evolution of plasticity: phytochrome-mediated shade avoidance responses. Integr. Comp. Biol. 43, 459–469. doi: 10.1093/icb/43.3.459, PMID: 21680454

[B69] Sharma A. K. Raghuram N. Chandok M. R. Das R. Sopory S. K. (1994). Investigations on the nature of the phytochrome-induced transmitter for the regulation of nitrate reductase in etiolated leaves of maize. J. Exp. Bot. 45, 485–490. doi: 10.1093/jxb/45.4.485

[B70] Sherameti I. Sopory S. K. Trebicka A. Pfannschmidt T. Oelmuller R. (2002). Photosynthetic electron transport determines nitrate reductase gene expression and activity in higher plants. J. Biol. Chem. 277, 46594–46600. doi: 10.1074/jbc.M202924200, PMID: 12244040

[B71] Shingles R. Roh M. H. McCarty R. E. (1996). Nitrite transport in chloroplast inner envelope vesicles (I. Direct measurement of proton-linked transport). Plant Physiol. 112, 1375–1381. doi: 10.1104/pp.112.3.1375, PMID: 12226452 PMC158066

[B72] Sugiura M. Georgescu M. N. Takahashi M. (2007). A nitrite transporter associated with nitrite uptake by higher plant chloroplasts. Plant Cell Physiol. 48, 1022–1035. doi: 10.1093/pcp/pcm073, PMID: 17566055

[B73] Suzuki A. Burkhart W. Rothstein S. (1996). Nitrogen effects on the induction of ferredoxin-dependent glutamate synthase and its mRNA in maize leaves under the light. Plant Sci. 114, 83–91. doi: 10.1016/0168-9452(95)04309-8

[B74] Suzuki A. Rioual S. Lemarchand S. Godfroy N. Roux Y. Boutin J. P.. (2001). Regulation by light and metabolites of ferredoxin-dependent glutamate synthase in maize. Physiol. Plant 112, 524–530. doi: 10.1034/j.1399-3054.2001.1120409.x, PMID: 11473712

[B75] Teller S. Schmidt K. H. Appenroth K. J. (1996). Ferredoxin-dependent but not NADH-dependent glutamate synthase is regulated by photochrome and a blue/UV-A light receptor in turions of *Spirodela polyrhiza* . Plant Physiol. Biochem. 34, 713–719.

[B76] Tollenaar M. (1989). Response of dry matter accumulation in maize to temperature: I. Dry matter partitioning. Crop Sci. 29, 1239–1246. doi: 10.2135/cropsci1989.0011183X002900050030x

[B77] Tsay Y. F. Chiu C. C. Tsai C. B. Ho C. H. Hsu P. K. (2007). Nitrate transporters and peptide transporters. FEBS Lett. 581, 2290–2300. doi: 10.1016/j.febslet.2007.04.047, PMID: 17481610

[B78] Umar A. S. Iqbal M. (2007). Nitrate accumulation in plants, factors affecting the process, and human health implications, A review. Agron. Sustain. Dev. 26, 45–57. doi: 10.1051/agro:2006021

[B79] Unkles S. E. Hawker K. L. Grieve C. Campbell E. I. Montague P. Kinghorn J. R. (1991). *crnA* encodes a nitrate transporter in *Aspergillus nidulans* . Proc. Natl. Acad. Sci. U.S.A. 88, 204–208. doi: 10.1073/pnas.88.1.204, PMID: 1986367 PMC50778

[B80] Van Gelderen K. Kang C. Li P. Pierik R. (2021). Regulation of lateral root development by shoot-sensed far-Red light via HY5 is nitrate-dependent and involves the NRT2.1 nitrate transporter. Front. Plant Sci. 12. doi: 10.3389/fpls.2021.660870, PMID: 33868355 PMC8045763

[B81] Vidal E. A. Alvarez J. M. Araus V. Riveras E. Brooks M. D. Krouk G.. (2020). Nitrate in 2020: Thirty years from transport to signaling networks. Plant Cell 32, 2094–2119. doi: 10.1105/tpc.19.00748, PMID: 32169959 PMC7346567

[B82] von der Fecht-Bartenbach J. Bogner M. Dynowski M. Ludewig U. (2010). CLC-b-mediated NO_3_ ^-^/H^+^ exchange across the tonoplast of *Arabidopsis* vacuoles. Plant Cell Physiol. 5, 960–968. doi: 10.1093/pcp/pcq062, PMID: 20430762

[B83] Warnasooriya S. N. Brutnell T. P. (2014). Enhancing the productivity of grasses under high-density planting by engineering light responses: from model systems to feedstocks. J. Exp. Bot. 65, 2825–2834. doi: 10.1093/jxb/eru221, PMID: 24868036

[B84] Weijschedé J. Martínková J. de Kroon H. Huber H. (2006). Shade avoidance in *Trifolium repens*: costs and benefits of plasticity in petiole length and leaf size. New Phytol. 172, 655–666. doi: 10.1111/j.1469-8137.2006.01885.x, PMID: 17096792

[B85] Weinig C. Delph L. F. (2001). Phenotypic plasticity early in life constrains developmental responses later. Evolution 55, 930–936. doi: 10.1554/0014-3820(2001)055[0930:ppeilc]2.0.co;2, PMID: 11430653

[B86] Wray J. L. Fido R. J. (1990). “Nitrate reductase and nitrite reductase,” in Methods in plant biochemistry, vol. 3 . Ed. Lea P. J. (London: Academic press), 241–256.

[B87] Xiang S. Wu S. Jing Y. Chen L. Yu D. (2021). Phytochrome B regulates jasmonic acid-mediated defense response against *Botrytis cinerea* in *Arabidopsis* . Plant Divers. 44, 109–115. doi: 10.1016/j.pld.2021.01.007, PMID: 35281129 PMC8897165

[B88] Xie X. Z. Xue Y. J. Zhou J. J. Zhang B. Chang H. Takano M. (2011). Phytochromes regulate SA and JA signaling pathways in rice and are required for developmentally controlled resistance to *Magnaporthe grisea* . Mol. Plant 4, 688–696. doi: 10.1093/mp/ssr005, PMID: 21357645

[B89] Yang X. Nian J. Xie Q. Feng J. Zhang F. Jing H.. (2016). Rice ferredoxin-dependent glutamate synthase regulates nitrogen-carbon metabolomes and is genetically differentiated between *japonica* and *indica* subspecies. Mol. Plant 9, 1520–1534. doi: 10.1016/j.molp.2016.09.004, PMID: 27677460

[B90] Yao X. Nie J. Bai R. Sui X. (2020). Amino acid transporters in plants: Identification and function. Plants (Basel) 9, 972. doi: 10.3390/plants9080972, PMID: 32751984 PMC7466100

[B91] Yoneyama T. Suzuki A. (2020). Light-independent nitrogen assimilation in plant leaves: Nitrate incorporation into glutamine, glutamate, aspartate, and asparagine traced by ^15^N. Plants (Basel) 9, 1303. doi: 10.3390/Fplants9101303, PMID: 33023108 PMC7600499

[B92] Zayed O. Hewedy O. A. Abdelmoteleb A. Ali M. Youssef M. S. Roumia A. F.. (2023). Nitrogen journey in plants: From uptake to metabolism, stress response, and microbe interaction. Biomolecules 13, 1443. doi: 10.3390/biom13101443, PMID: 37892125 PMC10605003

[B93] Zhu A. Ibrahim J. G. Love M. I. (2019). Heavy-tailed prior distributions for sequence count data: removing the noise and preserving large differences. Bioinformatics 35, 2084–2092. doi: 10.1093/bioinformatics/bty895, PMID: 30395178 PMC6581436

